# Stage-specific metabolic allocation: nutrient investment strategies during *Lysiphlebia japonica* Ashmead development

**DOI:** 10.3389/fnut.2025.1636519

**Published:** 2025-08-06

**Authors:** Tingting Zhou, Ningbo Huangfu, Li Wang, Junyu Luo, Jinjie Cui, Xiangzhen Zhu, Sumei Wan, Xueke Gao

**Affiliations:** ^1^College of Life Science and Technology, Tarim University, Xinjiang, China; ^2^Research Base of Zhengzhou University, State Key Laboratory of Cotton Bio-Breeding and Integrated Utilization, Institute of Cotton Research, Chinese Academy of Agricultural Sciences, Henan, Anyang, China; ^3^College of Agriculture, Tarim University, Xinjiang, China

**Keywords:** multi-omics, metabolites, fatty acid, parasitic wasps, growth periods

## Abstract

**Introduction:**

Parasitic wasps are key biological control agents that rely on precise nutrient allocation to regulate host exploitation and optimize their own development. Nutrients, particularly lipids and energy-related metabolites, play a critical role in shaping stage-specific growth and survival strategies in parasitic wasps.

**Methods:**

To analyze the allocation patterns of metabolite resources during development of parasitoid wasps, the multi-omics analysis was employed to systematically investigate nutrient dynamics across three growth periods in *Lysiphlebia japonica* Ashmead, a major parasitoid of cotton aphid (*Aphis gossypii* Glover).

**Results:**

Here, a total of 753 metabolites were detected by untargeted metabolomics, with numerous nutritionally critical compounds including amino acids, fatty acids and carbohydrates showed stage-specific variations. A total of 31 fatty acids (11 SFAs, 9 MUFAs, 11 PUFAs) were identified by targeted fatty acid detection, exhibiting a notable variation across development notably, PUFAs remained consistently dominant throughout all stages, suggesting their essential role in parasitoid growth. Correlation analysis further indicated that *α*-ketoglutaric acid and glutamic acid were functionally associated with fatty acids, serving as potential developmental biomarkers.

**Discussion:**

This study presented the first comprehensive metabolomic atlas of *L. japonica* development, uncovering nutrient allocation strategies that synchronize with its life cycle. By identifying key metabolites and fatty acids involved in its growth, our work provided a theoretical foundation for enhanced artificial rearing of parasitic wasps. Overall, these findings offered novel insights for translating omics data into practical applications, with significant theoretical and practical implications for developing improved biological control strategies.

## 1 Introduction

Parasitic wasps (Hymenoptera), known as outstanding natural enemy resources for biological control of pests, are diversity in species and quantity, as well as representative models for the study of parasitic regulation. Parasitoids exhibit a wide range of hosts, encompassing Lepidoptera, Coleoptera, and Diptera. Due to the unique parasitic characteristics and specialized developmental process (1, 2), the study of trophic regulatory relationship between parasitic wasps and their hosts has always been a focus. Numerous studies have demonstrated that parasitic wasp larvae meet their physiological and metabolic needs by actively manipulating host nutrient resources, particularly amino acids, fatty acids and carbohydrates (3–5). For instance, research has showed that parasitoid wasps *Leptopilina boulardi* regulates host lipid metabolism through gut microbiota (3), while *Cotesia vestalis* employs symbiotic virus *Bracovirus* (6) to fulfill the growth and development needs of its offspring. Another study suggested that *Cotesia chilonis* utilized amino acid nutrient resources of host *Chilo suppressalis* to support its developmental requirements (7). Yet, the dynamic changes in nutrient resource across different developmental stages of parasitic wasps remain poorly understood.

Metabolomics, serving as a potent tool for clarifying intricate biological processes (8), through quantitative analysis of all small-molecule compounds (e.g., amino acids, lipids, and nucleotides) in organisms under certain circumstances (9), the types, amounts and variations of metabolites along with different states were confirmed, thereby revealing the characteristics of metabolic pathways and life activities in organisms (10, 11). Typically, metabolomic approaches are categorized into three types: untargeted, targeted, and broadly targeted (12), while integrated untargeted and targeted metabolomics has grown increasingly common to pinpoint underlying metabolites due to its extensive scope and comprehensiveness (13–15). Current metabolomics has been widely applied in the study of insect physiology and biochemistry (16), behavior (17), growth and development (18), and insect-microbe symbiosis (19), promoting our understanding of insect nutrition, immunity (20), pathogen interactions (16, 20), and ecological adaptation (17, 21–24). Insects possess diverse metabolites that participate in species-specific biochemical pathways and play critical roles in their life cycles (25, 26). In addition, these metabolites and metabolic pathways evolve with varying developmental stages and habitats, enabling insects to adjust to the complex ecosystems (27, 28). Qualitative and quantitative analysis was conducted through metabolomics to screen out differentially expressed metabolites in insects, analyze metabolic pathways of metabolites, and study the multiple dynamic responses of metabolite levels, which is conducive to better revealing the physiological changes of insects and more accurately clarifying the response mechanisms of insects to the external environments. For instance, by metabolomics, it was found that 111 metabolites in *Aphis gossypii* were significantly changed after exposure to imidacloprid with different concentrations, which revealed the effects of neonicotinoid pesticides on aphids (29). By measuring the metabolome of *Drosophila melanogaster* under different temperature treatments, results showed that temperature exerted short-term and long-term effects on the metabolite concentration of *D. melanogaster* (30). Through quantitative analysis, it was reported that the metabolites of *Spodoptera exigua* altered notably after leaf feeding, including glucose and ferulic acid, providing new insights into plant-insect interaction (31). Likewise, comparative metabolomics of diapausing and non-diapausing *Exorista civilis* detected a total of 332 differentially abundant metabolites, among which L-proline is involved in multiple metabolic pathways of amino acid metabolism, suggesting that L-proline is crucial for maintaining life activities and resisting low temperature stress during diapause of *E. civilis* (32).

Fatty acids are the main energy source of insects and play critical roles in their growth and development, information exchange, and reproduction (33–35), which are classified into saturated fatty acids (SFA), monounsaturated fatty acids (MUFA), and polyunsaturated fatty acids (PUFA). The composition and content of fatty acids exhibit significant interspecific variation and influenced by multiple factors including sex, age, developmental stage, and environmental conditions (35, 36). For example, the total content of unsaturated fatty acids (UFAs) in most insects is higher than that of SFAs, among which C16:0 (palmitic acid), C18:1 (oleic acid) and C18:2 (linoleic acid) is predominant in SFAs, MUFAs and PUFAs, respectively (37). Likewise, it was found that the content of FAs in *Teleogryllus derelictus* gradually increased with growth and development, and there were notable differences between older nymphs, eggs and younger nymphs (38). Comparative analyses of FAs composition between herbivorous and omnivorous insects reveal distinct profiles that likely reflect species-specific metabolic adaptations and nutritional strategies, suggesting FAs composition is supposed to reflect metabolic and nutritional processes (39–41). Therefore, comparing amino acid and fatty acid contents in different growth periods of insects is of great significance to explore the metabolic mechanism of nutrients related to insect reproduction. Of which, the metabolism and fatty acids in parasitic wasps have received extensive attention due to their vital involvement in growth and development. For instance, Nurullahoglu et al. (42) discovered that the fatty acid composition within parasitoids is highly similar to that in their host aphids. Visser and Ellers (43) deduced that parasitoids, lacking enzymes for fatty acid synthesis, employ a tactic of directly exploiting the fatty acids in aphids for their biosynthetic metabolic processes to ensure their regular growth and development. Although the parasitic wasps continuously consumed host fat, the fatty acid concentration of *Achoria grisella*, which was parasitized by *Apanteles galleriae*, remained constant, and the overall fatty acid level of the wasps significantly exceeded that of the host.

Hence, it is interesting and valuable to study the composition and abundance changes of metabolites, especially fatty acids in parasitic wasps. So far, most of the metabolomics and FAs studies of parasitoids have focused on the environmental adaptation and host nutritional regulation, but the dynamic changes of parasitoids with their growth and development stages have not been fully studied. *Lysiphlebia japonica* (Ashmead) is the dominant parasitic wasp in northern cotton fields and has been used globally for the control of aphids, *A. gossypii* Glover. Here, integrated untargeted metabolomics and targeted FA analysis was employed to comprehensively profile metabolic composition and abundance across three key growth periods in *L. japonica* (larval, pupal and adult). Through differential and correlation analysis, the vital metabolites and FAs related to growth were obtained, and the accumulation rules during developmental periods were preliminarily explored. Results and strategies of this study not only advance our understanding of parasitoid biological traits, but also provide a scientific basis for guiding the artificial reproduction and utilization of parasitic wasps and developing improved biological control methods.

## 2 Materials and methods

### 2.1 Insects rearing

The initial generation of parasitoid *L. japonica* was collected from the cotton field at the Cotton Research Institute, Chinese Academy of Agricultural Sciences in Anyang (36°5’34.8”N, 114°31’47.19”E). Subsequently, the strain was reared in a laboratory setting with controlled conditions (26 ± 1°C, RH 75 ± 5%, L: D 14: 10), with which *A. gossypii* as a regular host. Aphids were reared on cotton leaves at 26 ± 1°C and 65% ± 5% relative humidity with a 14: 10 h L: D photoperiod. The second-instar aphids were exposed to the mated wasps for the purpose of obtaining the parasitized aphids. To exclude the potential influence of other conditions, the population was sustained until a minimum of 10 generations had been raised.

### 2.2 Sample collection

The samples used for metabolite extraction in this study were parasitic wasps at larval stage, pupal stage and adult stage, respectively. In a sterile environment, the 3-day parasitized cotton aphids were dissected and then the parasitic wasp larvae were carefully acquired. Samples were taken on the third day after transforming into pupae and on the third day after emergence. The adult insects were a mixture of both females and males. All samples were snap-frozen with liquid nitrogen immediately after collection or dissection. Three biological replicates (each replicate containing 30 samples from independent batches) were performed.

### 2.3 Metabolites extraction

Take 10 ± 1 mg sample into the 2 mL EP tubes, extracted with 600 μL extraction liquid (VMethanol: VChloroform = 3: 1), add 10 μL of L-2-Chlorophenylalanine (1 mg/mL stock in dH_2_O) as internal standard, vortex mixing for 30 s; Homogenized in ball mill for 4 min at 45 Hz, then ultrasound treated for 5 min (incubated in ice water); Centrifuge for 15 min at 12000 rpm, 4°C; Transfer the supernatant 500 μL into a fresh 1.5 mL EP tubes, take 30 μL from each sample and pooling as QC (Quality Control) sample. Dry completely in a vacuum concentrator without heating. Add 100 μL Methoxyamination hydrochloride (20 mg/mL in pyridine) incubated for 30 min at 80°C; Add 120 μL of the BSTFA regent (1% TMCS, v/v) to the sample aliquots, incubated for 1.5 h at 70°C; Add 8 μL FAMEs (in chloroform) to the QC sample when cooling to the room temperature. All samples were analyzed by gas chromatograph system coupled with a Pegasus HT time-of-flight mass spectrometer (GC-TOF-MS).

### 2.4 GC-TOF-MS analysis

GC-TOF-MS analysis was performed using an Agilent 7,890 gas chromatograph system coupled with a Pegasus HT time-of-flight mass spectrometer. The system utilized a DB-5MS capillary column coated with 5% diphenyl cross-linked with 95% dimethylpolysiloxane (30 m × 250 μm inner diameter, 0.25 μm film thickness; J&W Scientific, Folsom, CA, United States). A 1 μL aliquot of the analyte was injected in splitless mode. Helium was used as the carrier gas, the front inlet purge flow was 3 mL/min, and the gas flow rate through the column was 1 mL/min. The initial temperature was kept at 50°C for 1 min, then raised to 310°C at a rate of 10°C·min^−1^, then kept for 8 min at 310°C. The injection, transfer line, and ion source temperatures were 280, 280, and 250°C, respectively. The energy was −70 eV in electron impact mode. The mass spectrometry data were acquired in full-scan mode with the m/z range of 50–500 at a rate of 12.5 spectra per second after a solvent delay of 6.1 min.

### 2.5 Data preprocessing and annotation

Chroma TOF 4.3X software of LECO Corporation and LECO-Fiehn Rtx5 database were used for raw peaks extracting, the data baselines filtering and calibration of the baseline, peak alignment, deconvolution analysis, peak identification and integration of the peak area (44). Both of mass spectrum match and retention index match were considered in metabolites identification. Remove peaks detected in < 50% of QC samples or RSD > 30% in QC samples (45).

### 2.6 Free fatty acids detection

The samples used for fatty acid detection in this study were parasitic wasps at larval stage, pupal stage and adult stage, respectively. Free fatty acids detected by using a test kit and GC–MS analysis. For using a test kit, fatty acid extraction, detection, and analysis were performed according to the manufacturer’s instructions of the Free Fatty Acid Fluorometric Assay Kit (Cayman Chemical, United States) (46). For using GC–MS analysis, the specific steps are as follows.

### 2.7 Free fatty acids extraction

The samples were weighed into the 2 mL EP tubes, extracted with 1,000 μL (VIsopropanol: VHexane = 3: 2) as extraction liquid, and added 5 μL Trans C17 (1 mg/L) as internal standard. Homogenized in ball mill for 0.5 min at 1500 rpm, followed by ultrasound treated for 5 min, repeat 3 times. Centrifuge for 3 min at 14000 rpm, 4°C, then transfer 900 μL supernatant to the new tube. The tube was completely dried in a vacuum concentrator without heating, and 2 mL Methyl ester solution (Vmethanol: Vsulfuric acid = 15: 1) was added and incubated at 90°C for 1 h. Extracted with 2 mL Hexane twice, dried with nitrogen, and separated with 100 μL hexane at last. All samples were analyzed by gas chromatograph system coupled with a mass spectrometer (GC–MS).

### 2.8 GC–MS analysis

GC–MS analysis was performed using an SHIMADAZU 2010PLUS. The system utilized a SP2560 capillary column. A 1 μL aliquot of the analyte was injected in split mode (10: 1). Helium was used as the carrier gas, the front inlet purge flow was 5 mL/min, and the gas flow rate through the column was 1 mL/min. The initial temperature was 90°C hold on 1 min; raised to 170°C at a rate of 10°C/min, hold on 5 min; raised to 175°C at a rate of 5°C/min, hold on 0 min; raised to 210°C at a rate of 1°C/min, hold on 5 min; raised to 240°C at a rate of 5°C/min, hold on 20 min. The injection, transfer line and ion source temperatures were 250°C, 250°C and 300°C. The mass spectrometry data were acquired in scan mode with the m/z range of 50–727 after a solvent delay of 5 min (47).

### 2.9 Data analysis

For GC–MS the resulting absolute quantitative data were entered into the SIMCA14.1 software package (V14.1, Sartorius Stedim Data Analytics AB, Umea, Sweden) for principal component analysis (PCA) and orthogonal projections to latent structures-discriminant analysis (OPLS-DA). To determine significance, the changed fatty acids were assessed by the student’s t test (*p* < 0.05). The homogeneity of data was first tested by Bartlett’s test (R bartlett.test) (*p* < 0.05). In order to conduct a comparative analysis of metabolic differences between multiple experimental groups (*n* ≥ 3), we used one-way analysis of variance (one-way ANOVA, R language package one-way test) based on the mean distribution for multiple experiments. Statistical analysis and graph drawing were performed in GraphPad Prism version 9.0 (GraphPad Software).

## 3 Results

### 3.1 Quality control and PCA analysis of untargeted metabolomics

**Figure 1:**
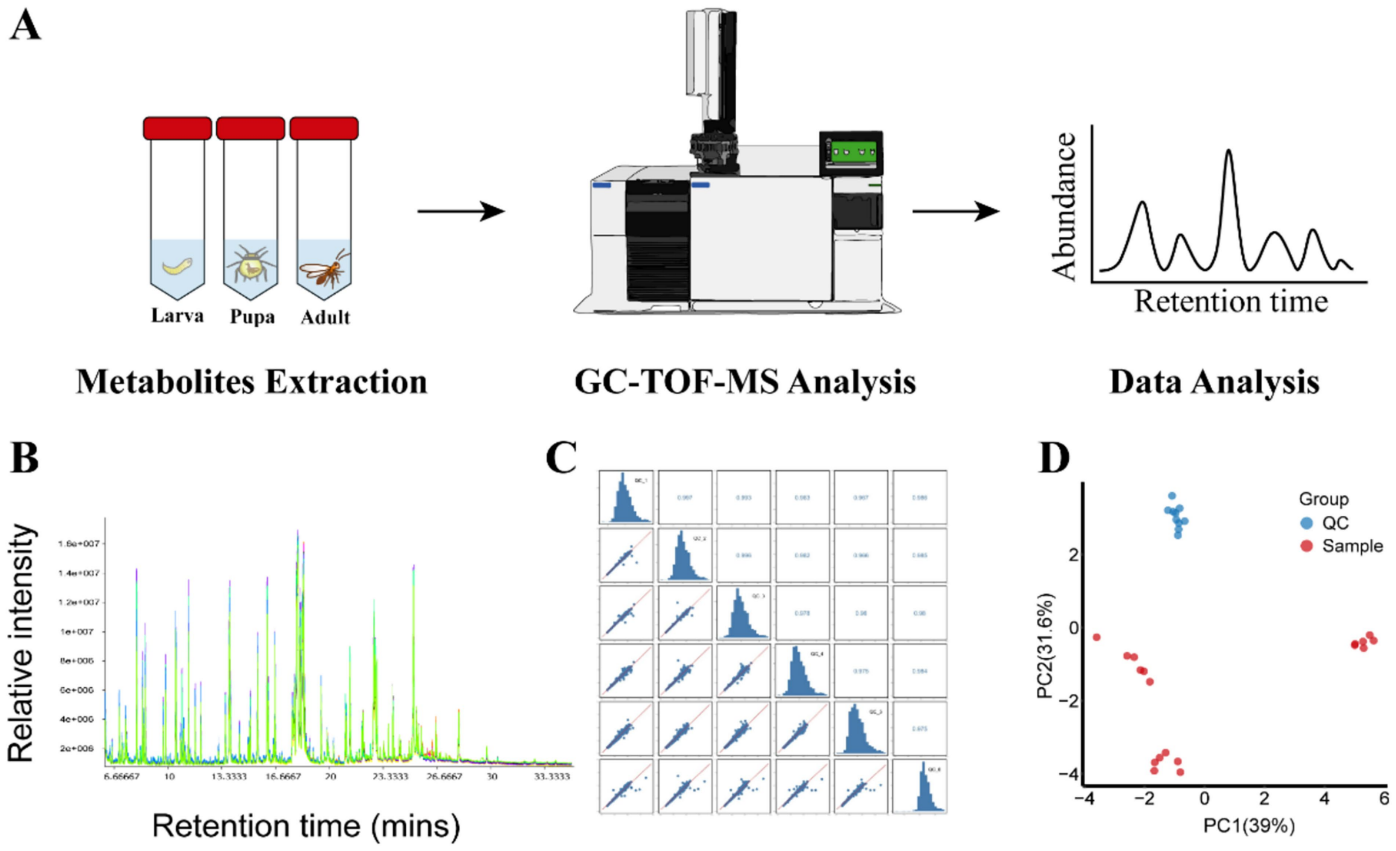
Metabolite profiling of wasp *Lysiphlebia japonica* and QC. **(A)** Process for GC-TOF-MS analysis of metabolites extraction from *L. japonica* during growth periods. **(B)** TIC stack of all QC samples. **(C)** Correlation heatmap of QC samples in *L. japonica*. **(D)** Score scatter plot for PCA model Sample with QC.

**Figure 2:**
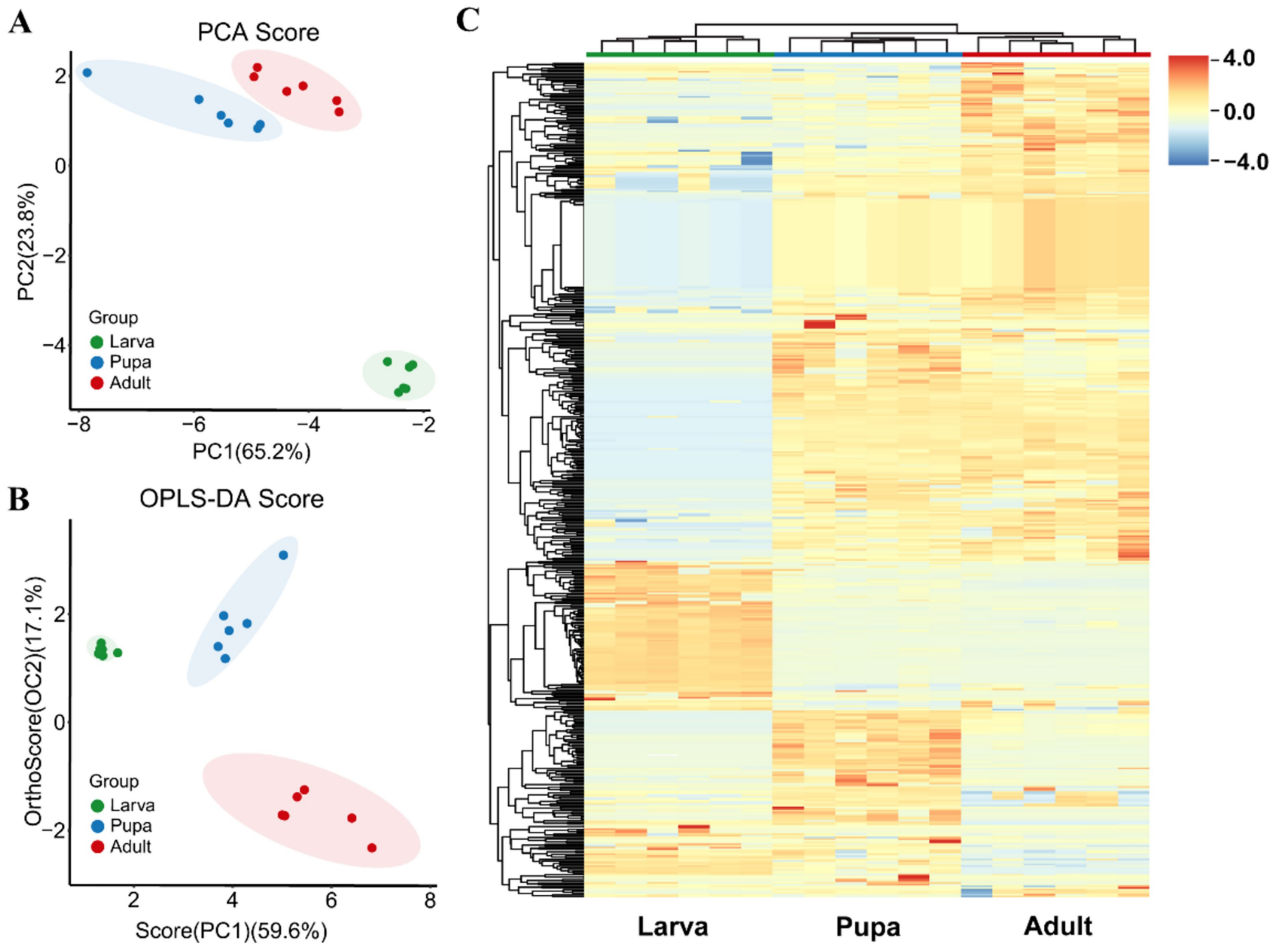
Metabolites accumulation during growth periods. **(A)** PCA score plots of *L. japonica* metabolites during growth periods. Green, blue and red spots represent the larval stage, pupal stage and adult stage, respectively. Percentages indicate the degree to which component explains the data set. **(B)** OPLS-DA score plots of *L. japonica* metabolites during growth periods. **(C)** Hierarchically heatmap of total 753 different metabolites during the three stages. Each column indicates one sample and row indicates metabolite. Red represents metabolites with high relative abundance which blue represents low relative abundance.

In this study, the metabolites of parasitic wasp *L. japonica* at three developmental stages-larva, pupa and adult-were quantified using GC-TOF-MS untargeted metabolomics method (Figure 1A). As shown in Figures 1B,C, there was a significant overlap between the retention time and the TIC peak area in QC samples, indicating the stability of instrument. Moreover, in the PCA analysis (Principal component analysis) diagram (Figure 1D), the QC samples were closely grouped, with a correlation coefficient exceeding 0.974, indicating good stability and high quality of the analysis system, and the obtained data were reliable. Therewith, PCA and OPLS-DA analysis (Orthogonal Projections to Latent Structures- Discriminant Analysis) were performed on all samples. In the study, the permutation test was employed to validate the PCA and OPLS-DA models. Findings revealed that the explanatory power (R2Y) and predictive power (Q2) of the samples were 0.993 and 0.94, respectively, indicating that the established model was consistent with the real situation of the data, the model was not overfitted. PCA results (Figure 2A) revealed that components 1 and 2 explained 65.2 and 23.8% of the metabolic differences, respectively, while OPLS-DA (Figure 2B) accounted for 59.6 and 17.1%, indicating a notable separation among larva, pupa, and adult stages. Besides, scattered points within the groups gathered together, suggesting a good repeatability in the group. Further findings revealed a closer proximity between pupa and adult on the PCA diagram, indicating that the metabolic profiles of the two periods were more similar.

### 3.2 Metabolites accumulation during growth periods

Across three different developmental phases of parasitic wasp *L. japonica*, in total of 753 metabolites, encompassing amino acids, fatty acids, organic acids, and amines, were detected. Following this, hierarchical cluster analysis and heatmap analysis were performed on 753 metabolites, with each column indicating the sample and each row the metabolite (Figure 2C). The results showed high reproducibility within the group, with metabolites distinctly isolated and exhibited a consistent pattern of alteration.

The hierarchical structure of metabolites is displayed on the left, divided from top to bottom into five unique groups. The primary metabolites of the first group are organic acids along with their derivatives like 2-deoxy-D-glucose, 4-aminobutyric acid 1, erythrose 2, lipoic acid, aminooxyacetic acid, and phosphate, predominantly found in adults. Metabolites of the second group consisted of amino acids and lipids, nucleosides and their derivatives, such as xanthine, inosine, oxalic acid, uric acid, linoleic acid, lactic acid, glycine, palmitic acid, stearic acid, and so on, of which was lower in larvae compared to pupae and adults. Conversely, the metabolites fond in the third group are most abundant in larvae, encompassing organic acids, lipids, carbohydrates, etc., such as proline, ribose, arachidic acid, lactose, glutamine, gluconic acid. The fourth group were highly expressed in pupae, which mainly include lipids (oleic acid, palmitoleic acid), amino acids (serine, L-cysteine, tyrosine, lysine, glycine) and carbohydrates (xylitol, maltotriose). The composition of the last group, contain 3-methylcatechol, sucrose, diglycerol, trehalose, and 2-ketovaleric acid, was more abundant in larvae and pupae than in adults. To sum up, metabolite levels of the 3rd group peaked during the larval phase, whereas the metabolite levels of the 1st, 4th and 5th group underwent notable alterations in both pupal and adult stages.

### 3.3 Differential metabolites during growth periods

**Figure 3:**
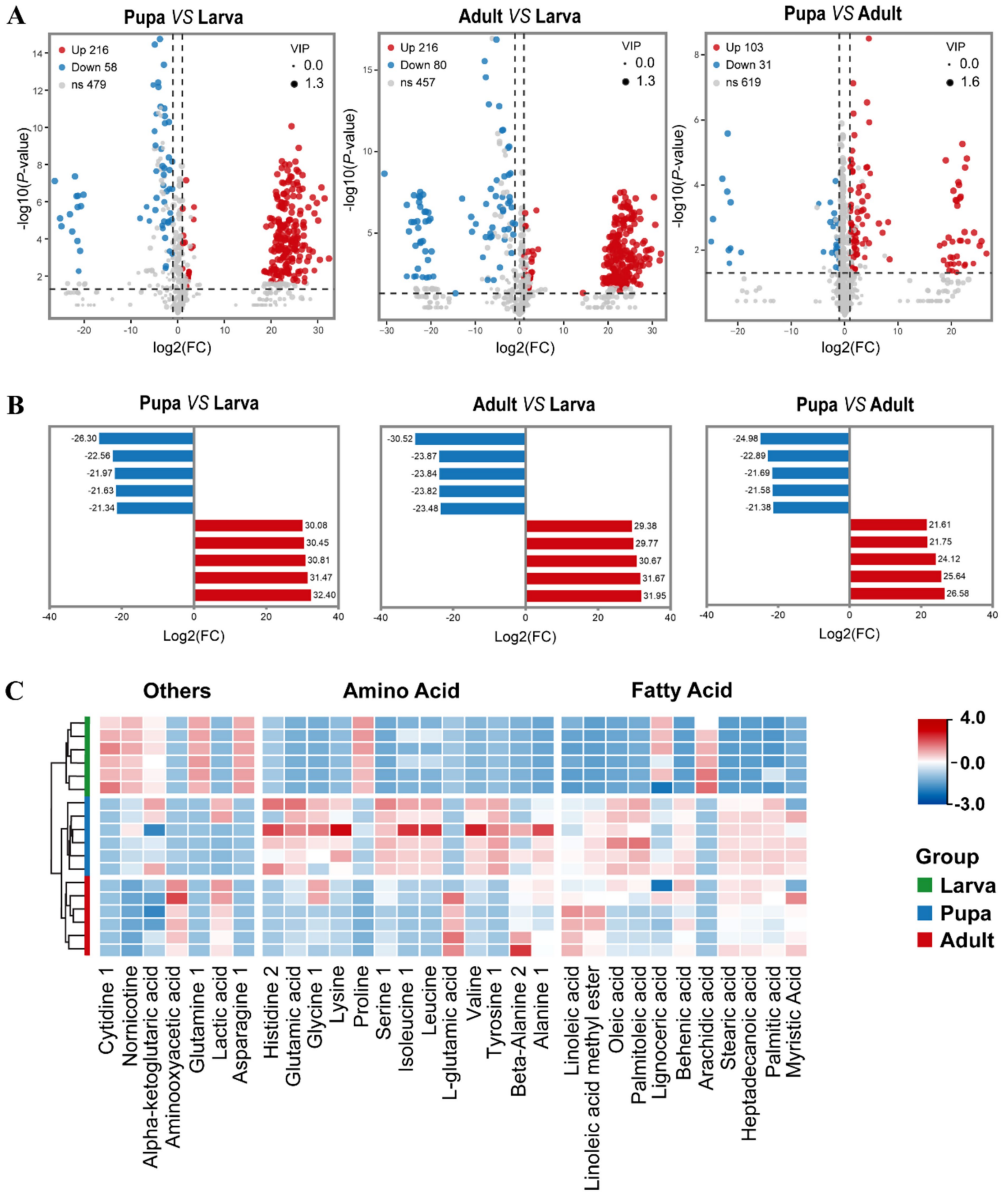
The profiles of differentially expressed metabolites when compared pair-to-pair. **(A)** Volcano plot showed the metabolites differentially expressed between Pupa and Larva, Adult and Larva, Pupa and Adult. Significantly up-regulated differentially expressed metabolites are shown in red, significantly down-regulated metabolites are shown in blue, and non-differentially expressed metabolites are shown in gray. | Log_2_FoldChange | > 1, *p* < 0.05 and VIP value > 1. **(B)** Histogram of top 5 up-accumulated and top 5 down-accumulated metabolites between Pupa and Larva, Adult and Larva, Pupa and Adult. Each column represents a metabolite, and the numbers are multiples of the difference, with red indicating up-regulation and blue indicating down-regulation. **(C)** Heatmap of fatty acids and amino acids during three stages.

Following this, we took multivariate statistical methods to screen the identified 753 metabolites, aiming to pinpointing those with the most significant differences among larvae, pupae, and adults of *L. japonica*. Under the condition of VIP (Variable Importance in the Projection) > 1, *p* < 0.05, along with │log_2_FC│ > 1, a total of 381 differentially expressed metabolites were analyzed, mainly amino acids, fatty acids, and carbohydrates, highlighting their predominant contribution to the difference. Figure 3A showed the volcanic map of differential metabolites in the three growth periods. Subsequently, the five highest and lowest log_2_FC values from each group were selected to perform the bar chart of differential multiples (Figure 3B). The results showed that compared with the larval phase, there were 274 differentially expressed metabolites in the pupal stage, with 216 up-regulated (indicated by red spots in Figure 3A) and 58 down-regulated (marked by blue spots in Figure 3A), showing significant differences (*p* < 0.001). Among them, amino acids like alanine (log_2_FC = 32.4), tyrosine, and valine had the highest upregulation times, with lipids like stearic acid and oleic acid trailing behind. As for the most significant decrease in expression, organic acids and derivatives, including glutamine (log_2_FC = −26.3), and 3-hydroxypyruvate, exhibited the greatest downregulation ratio, followed by nucleotides and derivatives such as cytidine 1. Compared with larval stage, 216 metabolites were significantly up-regulated in adults, among which alanie, stearic acid, lactic acid had the highest upregulation ratio, and asparagine, Allylmalonic acid had the highest downregulation ratio. As for the comparison between pupa and adult, volcanic map indicated that 103 metabolites were up-regulated, 31 were down-regulated, and VIP was up to 1.6. Besides, the difference of histidine2 was the largest among up-regulated metabolites (26.58), and L-glutamic acid had the smallest difference multiple (−24.98) among down-regulated metabolites.

On this basis, our attention was centered on the alterations in fatty acids and amino acids within *L. japonica* throughout their developmental stages. Figure 3C illustrated that the majority of fatty acids are minimal during the larval phase and built up significantly in the pupal phase, like glutamic acid, serine, tyrosine, oleic acid, and stearic acid, potentially linked to the unique growth conditions of parasitic wasps. Most amino acids were present in low amounts during the larval phase, then plentiful in the pupal and gradually reduced in the adult stage. Among them, the levels of arachidic acid, lignoceric acid, and proline peaked in the first stage of larva, progressively diminishing in the following two stages. Other metabolites, including *α*-ketoglutaric acid, cytidine 1, glutamine 1, and asparagine 1, exhibited comparable patterns.

### 3.4 Overview of fatty acid detection by GC–MS

**Figure 4:**
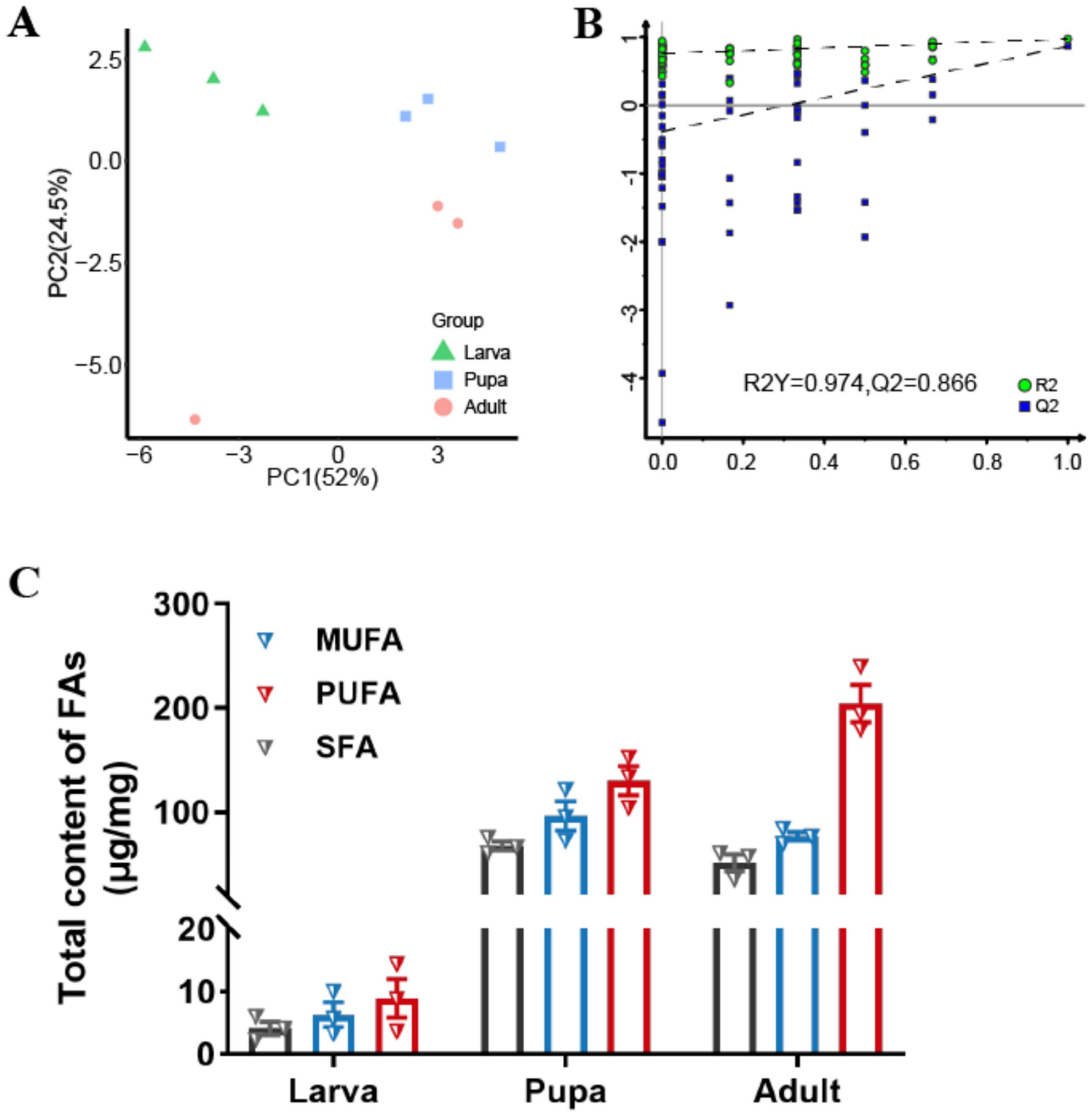
Fatty acids accumulation during growth periods. **(A)** Score plot of the PCA models applied to three growth stages. **(B)** Score plot of the OPLS-DA models applied to three growth stages. **(C)** Changes of total contents (μg/mg) of SFAs, MUFAs and PUFAs in different growth stages of *L. japonica*.

By further analyzing the fatty acid composition of *L. japonica* at different developmental stages, a complete list of detected fatty acids through GC–MS were obtained (Table 1). A total of 31 types of fatty acids were detected in this study, all of which belonged to the long-chain fatty acids, encompassing 11 saturated fatty acids (SFAs), 9 monounsaturated fatty acids (MUFAs), and 11 polyunsaturated fatty acids (PUFAs), with their overall content being PUFAs (353.45μg/mg) > MUFAs (179.41 μg/mg) > SFAs (122.70 μg/mg). Variations were observed in the contents of total fatty acids across species, as linoleic acid > vaccenic acid > stearic acid > palmitic acid > oleic acid, showing significant differences (*p* < 0.001). Principal component analysis revealed that the first and second principal components explained 76.5% of the total variance, effectively differentiating the three developmental stages and highlighting notable disparities in fatty acid content (Figure 4A). Meanwhile, findings of OPLS-DA revealed that the explanatory power (R2Y) and predictive power (Q2) of the samples were 0.974 and 0.866, respectively, indicating the consistency of the established model and the reliability of the results (Figure 4B).

**Table 1:** List of fatty acids and total content during growth periods.

No.	Category	Abbreviation	Fatty acid species	Total content(μg/mg)
1	SFA	C12:0	Laurate acid	2.71c
2	SFA	C14:0	Myristic acid	14.69c
3	MUFA	C14:1 T	Transmyristelaudate acid	1.29c
4	MUFA	C14:1	Myristoleate acid	0.96c
5	SFA	C15:0	Pentadecanoate acid	0.46c
6	SFA	C16:0	Palmitic acid	48.47bc
7	MUFA	C16:1	7-palmitoleic acid	15.05c
8	SFA	C17:0	Heptadecanoate acid	1.06c
9	MUFA	C17:1	10-heptadecenoate	0.45c
10	SFA	C18:0	Stearic acid	51.76bc
11	MUFA	C18:1	Oleic acid	19.44c
12	MUFA	C18:1–2	Vaccenic acid	141.09b
13	PUFA	C18:2TT	Linoelaidate acid	0.15c
14	PUFA	C18:2	Linoleic acid	328.65a
15	PUFA	C18:3	Gammalimolenate acid	0.144c
16	PUFA	C18:3–2	Alphalinolenate acid	4.82c
17	SFA	C20:0	Arachidate acid	2.44c
18	MUFA	C20:1	8-eiciseniate acid	0.88c
19	PUFA	C20:2	Eicosadienoate acid	0.33c
20	SFA	C21:0	Heneicosanoate	0.02c
21	PUFA	C20:3	11–14-17 eicosatrienoate	0.12c
22	MUFA	C22:1	Erucate acid	0.05c
23	SFA	C22:0	Behenate acid	0.33c
24	MUFA	C22:1 T	Brassidate acid	0.19c
25	SFA	C23:0	Tricosanoate acid	0.48c
26	PUFA	C20:4	Arachidonate acid	0.20c
27	PUFA	C22:2	Docosadienoate acid	18.47c
28	PUFA	C20:5	Eicosapentaenoate acid	0.19c
29	SFA	C24:0	Lignocerate acid	0.28c
30	PUFA	C22:5	Docosapentaenoatem	0.22c
31	PUFA	C22:6	Docosahexaenoate acid	0.17c
	SFAs	Total contents (μg/mg)		122.70
	MUFAs			179.41
	PUFAs			353.45

Different lowercase letters indicate significant differences among treatments at *p* < 0.05.

Fatty acid levels exhibited a certain trend of alteration correlating with the growth period of *L. japonica* (Figure 4C). The contents of SFAs and MUFAs generally rose initially and then decreased, in contrast to PUFAs, which saw a complete increase. During the larval phase, the levels of the three types of FAs were minimal, whereas in the pupa phase, SFAs and MUFAs peaked, and PUFAs were at their highest in the adult phase. Across all three periods, the content of PUFAs consistently exceeded those of SFAs and MUFAs, suggesting their significant contribution to the growth and development of parasitic wasps.

### 3.5 Fatty acids accumulation during growth periods

**Figure 5:**
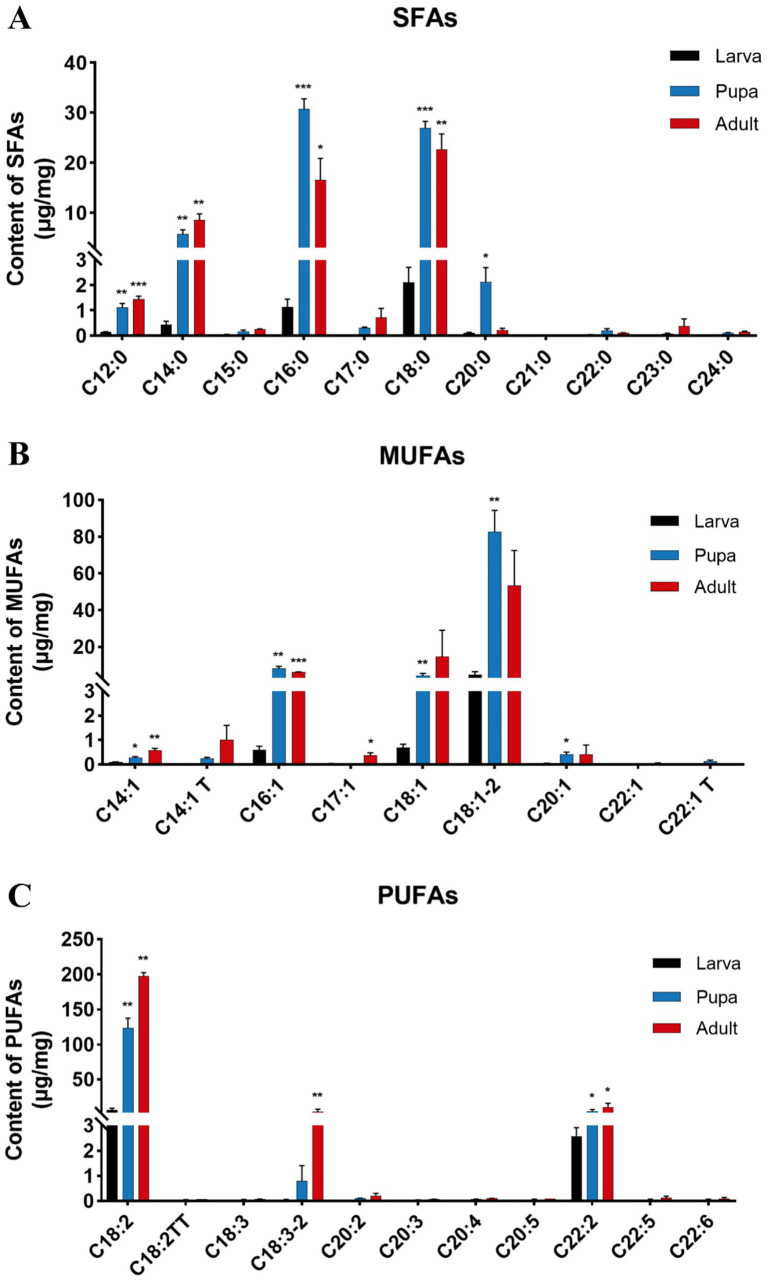
Changes of components and contents (μg/mg) of FAs in different growth stages of *L. japonica*. **(A)** 11 SFAs, **(B)** 9 MUFAs, and **(C)** 11 PUFAs. *p < 0.05, **p < 0.01, ***p < 0.001, ns: no significant.

We further analyzed the contents of various fatty acids to pinpoint those that might operate throughout different growth stages of *L. japonica* (Figure 5). Not surprisingly, the levels of 11 SFAs were lowest during larval stage, yet increased in both pupal and adult stage, with the majority exhibiting significant (*p* < 0.05) or extremely significant (*p* < 0.001) differences (Figure 5A). Within group of SFAs, C16:0 (palmitic acid) exhibited the highest content in pupal stage (30.78 μg/mg), while C18:0 (stearic acid) peaked in both larval (2.12 μg/mg) and adult stage (22.68 μg/mg), signifying their significant impact on the growth and development of *L. japonica.* Compared with the larval stage, the content of C16:0 in pupal and adult stage was increased by 27.3 times and 14.7 times, respectively, while C18:0 was 12.7 and 10.7 times, and C14:0 (myristic acid) was increased by 5.28 μg/mg and 8.12 μg/mg.

Of all the identified MUFAs, the contents of C18:1–2 (vaccenic acid) were the most elevated across the three phases of *L. japonica* (Figure 5B), measured at 4.76 μg/mg, 82.76 μg/mg and 53.57 μg/mg, in that order. Concurrently, the levels of C18:1–2 in the pupal stage was 17.40 times higher than that in the larval stage, reaching an extremely significant level (*p* < 0.01), highlighting its critical role during growth and development of *L. japonica*. With the growth period, the contents of C18:1–2 and C16:1 (7-palmitoleic acid) increased first and then decreased, whereas C14:1 T (transmyristelaudate acid) and C14:1 (myristoleate acid) increased successively.

In parasitic wasps *L. japonica*, PUFAs stood out as the predominant fatty acid species, exhibiting a remarkably consistent variation trend across different periods, showing a pattern of adult stage > pupal stage > larval stage, with significant or extremely significant alterations in C18:2 (linoleic acid), C18:3–2 (alphalinolenate acid), and C22:2 (docosadienoate acid). Among the group of PUFAs, the total content of C18:2 was the highest (328.65 μg/mg), indicating its indispensable role in the growth of *L. japonica*. When compared with larval stage, C18:2’s contents in pupal and adult stages showed increases of 116.91 μg/mg and 191.08 μg/mg, respectively, which aroused our attention as well (Figure 5C).

### 3.6 Correlation analysis

To further elucidate the mutual regulatory relationships between metabolite expression patterns and fatty acids accumulation during the growth and development of *L. japonica*, we then conducted correlation analysis between all significantly altered metabolites and FAs (VIP > 1, *p* < 0.05) by using the Spearman algorithm. Fatty acids and metabolites with expression correlation may jointly participate in a biological process, that is, functional correlation. In this study, metabolites positively correlated with free fatty acids were *α*-ketoglutaric acid, glutamic acid (S)-Mandelic acid, alpha-ketoisocaproic acid 1, gluconic acid 1, succinate semialdehyde 2, maltotriose 2, proline, et al. While metabolites negatively correlated with fatty acids include Atropine, Glucoheptonic acid 1, maltose, Loganin, palatinitol 2, xylitol, etc. The detailed correlation data are shown in Supplementary Table S1.

## 4 Discussion

Lately, parasitic wasps have received high attention owing to their remarkable efficacy in biological pest control. While current metabolomic and fatty acid research on parasitoids has primarily concentrated on adapting to environmental adaptation and host nutritional regulation, nonetheless, the dynamic alterations in metabolite composition and abundance during growth and development stages are still unclear. In this study, we employed an integrated approach combining untargeted metabolomics and targeted fatty acid analysis to profile metabolic changes in the parasitic wasp *L. japonica* across three key growth periods. Our analysis detected a total of 753 metabolites, with differentially abundant metabolites predominantly belonging to amino acids, fatty acids and carbohydrates. Additionally, we characterized the growth period-specific variations in 31 types of fatty acids and their contents. These findings provide valuable insights into the metabolic basis of parasitoid growth and development, and offer important implications in developing guidance for the artificial breeding of parasitic wasps.

There are various metabolites in organisms such as plants and animals, and the metabolites of most species vary with sex and age, and the differences between metabolomes show a tendency to increase with age (24, 48–50). Based on untargeted metabolomics, we constructed a dynamic map of metabolic accumulation at the stages of larvae, pupae and adults of parasitic wasp *L. japonica*, finding that there were significant differences in metabolites during the growth and development, which also provided evidence for previous studies (51–53). In contrast to the pupal and adult stages, larval stage metabolites show notable differences, yet the metabolites at pupal and adult stages remain largely unaltered. Via cluster analysis, we identified that a large number of metabolites, such as *α*-ketoglutaric acid, proline, ribose, isocitric acid and gluconic acid accumulated in the larval stage, and then gradually decreased with the growth stage (Figure 2C), most of which were carbohydrates and organic acids. As energy reserve and nutrients for organisms, carbohydrates play an important role for the growth and development of insect larvae, which can promote the proliferation and rapid growth of larval tissue cells as well (54–56). Amino acids and fatty acids are essential energy and substances in controlling the metabolism and growth of insects (57, 58), especially for maintaining the survival of the parasitoid pupal stage and the development of adult tissues. Similarly, findings in this study revealed that large quantities of amino acids and fatty acids were enriched in the pupal and adult stages (Figure 2C), including serine, glutamic acid, tyrosine, lysine, glycine, and linoleic acid, lactic acid, palmitic acid, stearic acid, and oleic acid.

With function of increasing larva autophagy rate via directly inhibits ATP synthase (57, 59, 60), *α*-ketoglutaric acid is an important metabolite in a majority of biological process (57, 61). Glutamic acid is a vital amino acid and involved in promoting adult cell proliferation (62), and deemed to be one of the major energy sources in the pupa-adult transition, during which the insect undergoes metamorphosis and also loses its food supply (57). In our study, alpha-ketoglutaric acid was detected highly expressed in larval stage, while glutamic acid was found at higher levels in pupal than the other stages, which were regarded as a marker metabolite of pupal stage. In addition, correlation analysis results showed that α-ketoglutaric acid and glutamic acid, were positively correlated with fatty acids, indicating that they were functionally related, that is, they were highly likely to participate in the growth and development of parasitic wasps. Alpha-ketoglutaric acid and glutamic acid can be converted to each other by corresponding enzymes. The glutamic acid *in vivo* is derived from ingested food and converted into α-ketoglutaric acid by enzymes, resulting in an increase in α-ketoglutaric acid abundance in the larval stage. Soon afterwards, alpha-ketoglutaric acid is transformed into glutamic acid by GDH-like (glutamate dehydrogenase-like), thereby raising the amount of glutamic acid in the pupal stage and providing sufficient energy for adult growth and development afterwards (63).

Fatty acids, as the basic unit of lipids, are abundant *in vivo* and play an important role in the physiological processes of insect growth, development, reproduction and adaptation to the environment (64). Our results showed that a total of 31 fatty acids were detected accompanying larval, pupal and adult stages of *L. japonica*, 11 saturated fatty acids (SFAs), 9 monounsaturated fatty acids (MUFAs) and 11 polyunsaturated fatty acids (PUFAs) are included. Most insects contain more unsaturated fatty acids than saturated fatty acids, some as much as 2.5 times higher (65, 66). Similarly, we observed that the contents of MUFAs and PUFAs were both higher than SFAs, 1.46 times and 2.88 times of SFAs, respectively. C18:2, C18:0, C16:0, C18:1–2 and C18:1 were the dominant FAs in the growth of parasitic wasps *L. japonica*, which was consistent with most conclusions (42, 67, 68). Nevertheless, C18:1–2 accounts for a large proportion of MUFAs in *L. japonica*, which is inconsistent with other insect orders. In our data, the content of C18:2 in PUFAs was the highest (328.65 μg/mg), while contents of C18:0 and C16:0 were dominant in SFAs, but there was no significant difference between them, which is consistent with the results of previous studies (42).

The accumulation and composition of fatty acids are closely related to the development, reproduction and viral infection of insects, and there are obvious differences in different survival environment as well. In this study, the contents of FAs showed an overall increasing trend with the growth period, which is consistent with Shi et al. (38) Compared with the other two stages, the larval stage had the lowest contents of SFAs, MUFAs and PUFAs, which we speculated was related to the parasitic characteristics of larva parasitic wasps and their inability to synthesize fatty acids. Parasitoids are generally believed to lack the enzyme system associated with fatty acid synthesis (43, 69), so they directly utilize or feed on host FAs in order to satisfy their own growth and development (70, 71). With the development of the growth period, the nutritional requirements gradually expanded, and the fatty acid content of parasitic wasps gradually increased, and SFAs and MUFAs reached their peak value in the pupal stage. The pupa is in the transition stage from larva in vegetative stage to adult in reproductive stage, and many nutrients such as amino acids and fatty acids are abundant in the pupa stage. Meanwhile, we found that PUFAs were consistently dominant in the three periods, and the content varied significantly in each period, which suggested to be related to the function of PUFAs. PUFAs not only play a crucial role in determining the structure and function of biofilms (72), but also is an important source of prostaglandins, which contribute to the development regulation (65). Moreover, PUFAs varied widely due to developmental stages, species, and external environment of vertebrates (36, 73). Our results showed that the contents of SFAs and MUFAs decreased after the emergence of parasitic wasps, while the contents of PUFAs increased all the way and reached a peak in the adult stage. Studies have shown that PUFAs affect the reproductive performance of animals through anabolic steroids, not only promote the development of animal reproductive organs, but also improve the ovulation rate and birth rate (74). Another study confirmed that PUFAs play an important role in improving ovarian function and promoting egg laying in *D. melanogaster* (75). Therefore, we speculate that the accumulation of PUFAs may be related to the occurrence of sexual reproductive activities in adult stage. Our results provide a theoretical basis for this conjecture as well. Since the content of UFAs in insects is higher than that in other animals, these studies provide new insights into better utilization of UFAs in insects and control of pests.

## 5 Conclusion

In conclusion, through multi-omics analysis, this study systematically characterized the dynamic accumulation of 753 metabolites and 31 fatty acids during development of parasitic wasp *L. japonica*, revealing the allocation patterns of metabolite resources. Key carbohydrates and organic acids dominated larval growth, while amino acids and fatty acids were crucial for pupal development. Differential analysis identified the potential key growth-related metabolites and FAs, while PUFAs, particularly linoleic acid (C18:2) maintained consistently high levels throughout developmental periods, suggesting their essential role in growth and reproductive processes. Correlation analysis further revealed functional associations between fatty acids and critical metabolites, particularly *α*-ketoglutaric acid and glutamic acid, which were considered as potential developmental stage biomarkers. Our study deepens insights into the growth and development traits of parasitic wasps, and offer a theoretical foundation for steering the artificial breeding and utilization of parasitic wasps in pest control strategies. By bridging omics with practical biocontrol needs, future studies should validate these metabolic biomarkers under operational settings to further improve biological control strategies, thereby maximizing the ecological potential of parasitoids in sustainable agriculture.

## Data Availability

The original contributions presented in the study are included in the article/Supplementary material, further inquiries can be directed to the corresponding author/s.
